# Comprehensive Analysis of Prognostic Value and Immune Infiltration of the NT5DC Family in Hepatocellular Carcinoma

**DOI:** 10.1155/2022/2607878

**Published:** 2022-01-10

**Authors:** Rongqi Li, Rongqiang Liu, Shiyang Zheng, Wenbin Liu, Hui Li, Dewei Li

**Affiliations:** ^1^Department of Hepatobiliary Pancreatic Tumor Center, Chongqing University Cancer Hospital, Chongqing 400030, China; ^2^Department of Hepatobiliary Surgery, Foshan Hospital of Traditional Chinese Medical, Foshan 528000, Guangdong, China; ^3^Department of Head and Neck Surgery, The Affiliated Cancer Hospital and Institute of Guangzhou Medical University, Guangzhou 510095, Guangdong, China

## Abstract

**Background:**

Hepatocellular carcinoma (HCC) is one of the most common malignant tumors in the world, and its incidence is obviously increasing. The NT5DC family has been shown to be involved in the progression of many tumors. However, the biological function of NT5DC family members in HCC is still not well understood.

**Methods:**

Oncomine, Gene Expression Profiling Interactive Analysis (GEPIA), UALCAN, Kaplan–Meier plotter, cBioPortal, GeneMANIA, Metascape, and TIMER were applied to assess the biological function of NT5DC family members in HCC.

**Results:**

Most of the NT5DC family members were highly expressed in HCC. High expression of NT5C2, NT5DC2, and NT5DC3 was closely associated with higher tumor stage and poor overall survival (OS). In addition, high NT5DC2 and NT5DC3 expression also predicted poor disease-free survival (DFS). Enrichment analysis revealed that the NT5DC family in HCC mainly involved the IMP metabolic process, purine ribonucleoside monophosphate metabolic process, and purine nucleoside monophosphate metabolic process. The expression of NT5DC family members was closely related to the infiltration of some immune cells, such as B cells, CD8+ T cells, CD4+ T cells, macrophages, neutrophils, and dendritic cells.

**Conclusion:**

Our findings provided new insights into the biological function and prognostic value of NT5DC family members in HCC.

## 1. Introduction

Hepatocellular carcinoma (HCC) is one of the most common malignant tumors in the world and the second leading cause of cancer death [1]. HCC accounts for about 90% of primary liver malignancies [2]. Data have shown that the incidence of HCC will continue to rise and peak around 2030 [3]. The treatment of HCC mainly includes hepatectomy or liver transplantation, supplemented by radiotherapy, radiofrequency ablation, and transarterial chemoembolization. The onset of HCC is insidious, and the early symptoms are not obvious. Most patients are in the middle and late stages when they are diagnosed, and the 5-year survival rate of patients after hepatectomy is still poor due to their high degree of malignancy [4]. Many prognostic markers have been applied for HCC, but the effect is not very satisfactory [5]. Therefore, a simple and effective prognostic marker is urgently needed to better predict the prognosis of HCC patients, so that clinicians can implement individualized treatment as soon as possible.

The NT5DC family is a kind of evolutionary conserved 5′-nucleotidase, which can catalyze the hydrolysis of nucleotides in cells [6]. The NT5DC family members include NT5C2, NT5DC1, NT5DC2, NT5DC3, and NT5DC4, and they all contain a haloacid dehalogenase motif localized in the N-terminus of these proteins [6]. Some previous studies suggested that the NT5DC family was associated with some psychiatric disorders [7]. Recently, the NT5DC family has been considered to play an important role in tumor progression [8, 9]. Currently, only a few studies have investigated the association between NT5DC family members and HCC. Chen et al. found that NT5DC2 was highly expressed in liver cancer tissues, and the overexpression of NT5DC2 indicated poor overall survival (OS) and relapse-free survival (RFS) [10]. Li et al. showed the overexpression of NT5DC2 promoted liver cancer cell proliferation through stabilizing epidermal growth factor receptor (EGFR) [11]. However, the prognostic value and biological function of NT5DC family members in HCC was still well understood. Therefore, we used bioinformatics methods based on different databases to conduct a comprehensive analysis of the potential value of the NT5DC family in HCC.

## 2. Materials and Methods

### 2.1. Oncomine

Oncomine is a publicly accessible online database with a large amount of tumor microarray data and can provide extensive and comprehensive genome-wide expression analysis [12]. The mRNA expression of the NT5DC family in HCC was identified by the Oncomine database. Student's *T*-test was used to analyze the difference of NT5DC family expression in HCC. The parameters were set as *P* value <0.01, fold change >2, gene rank: 10%, data type: mRNA; and analysis type: cancer vs. normal analysis.

### 2.2. UALCAN

UALCAN is a fast and effective online analysis and mining website, mainly based on the TCGA database related cancer data, and can provide a large number of comprehensive analysis, including gene expression, survival analysis, and epigenetic regulation [13]. In our study, we used UALCAN to evaluate the expression of the NT5DC family and its effect on liver cancer tumor staging. The *T*-test was used, and *P* value <0.05 was considered statistically significant.

### 2.3. GEPIA

GEPIA is a newly developed online database that can implement quick comprehensive analysis of TCGA database data [14]. We used GEPIA to analyze the relationship between NT5DC family expression and the prognosis of HCC patient.

### 2.4. Kaplan–Meier Plotter

The Kaplan–Meier plotter database integrates gene expression and clinical prognosis information for survival analysis or meta-analysis of a variety of tumors [15]. We further used the Kaplan–Meier plotter website to evaluate the prognostic value of NT5DC family members in HCC.

### 2.5. cBioPortal

cBioPortal is an online database that provides multidimensional cancer genomics analysis [16]. We used cBioPortal to assess gene mutations and copy number variation (CNV) of NT5DC family members in HCC.

### 2.6. GeneMANIA

GeneMANIA is a website for generating hypotheses about gene function, analyzing gene lists, and prioritizing genes for functional assays [17]. We used GeneMANIA to explore the relationship between NT5DC family members and their related proteins.

### 2.7. Metascape

Metascape is an online website tool that can provide multiple functions such as enrichment analysis and protein interaction network analysis [18]. We used it for enrichment analysis of the NT5DC family.

### 2.8. TIMER

TIMER is a visualization website that can analyze immune cell infiltration in different tumors [19]. We used it to evaluate the association between the expression of NT5DC family members and immune cell infiltration in HCC.

## 3. Results

### 3.1. The Expression of the NT5DC Family in HCC

**Figure 1:**
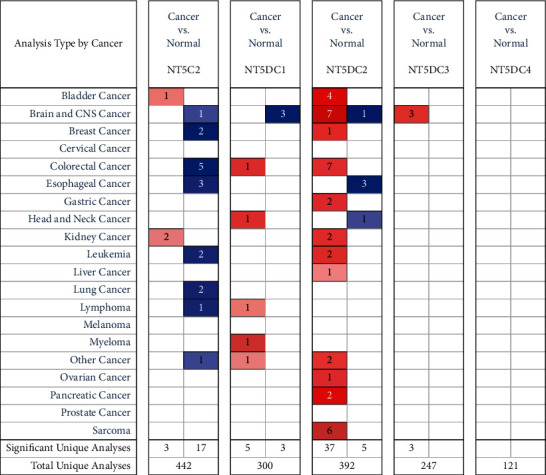
The mRNA expression of NT5DC family members in different tumors (Oncomine). Blue means downregulation, and red means upregulation.

**Figure 2:**
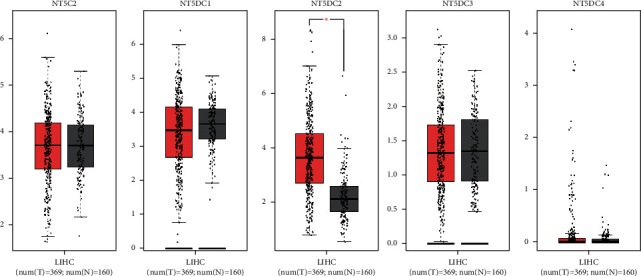
The mRNA expression of NT5DC family members in HCC patients (GEPIA).

**Figure 3:**
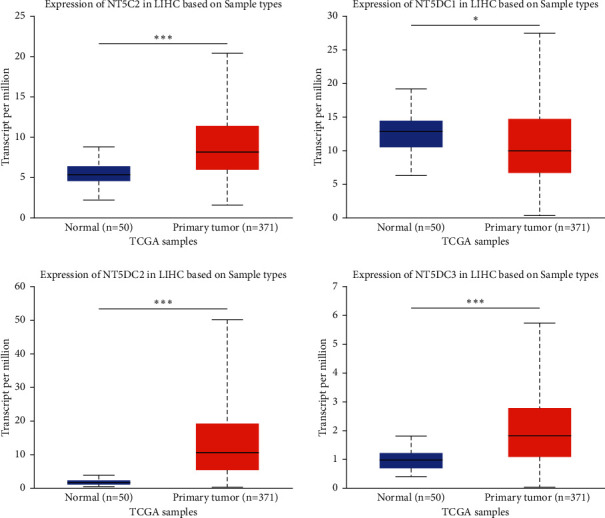
The protein expression of NT5DC family members in HCC patients (UALCAN).  ^*∗*^*P* < 0.05;  ^*∗*^ ^*∗*^ ^*∗*^*P* < 0.001.

Oncomine was used to evaluate the mRNA expression of NT5DC in HCC. The results showed that the transcriptional levels of NT5DC2 were significantly expressed in HCC (Figure 1). Furthermore, we used GEPIA to analyze the transcription of the NT5DC family. Similarly, we found that NT5DC2 was significantly expressed in HCC (Figure 2). Next, UALCAN was applied to determine the protein expression level of the NT5DC family in HCC. The results showed that NT5C2, NT5DC2, and NT5DC3 proteins were highly expressed in HCC (Figure 3).

### 3.2. Prognostic Significance of the NT5DC Family in HCC

**Figure 4:**
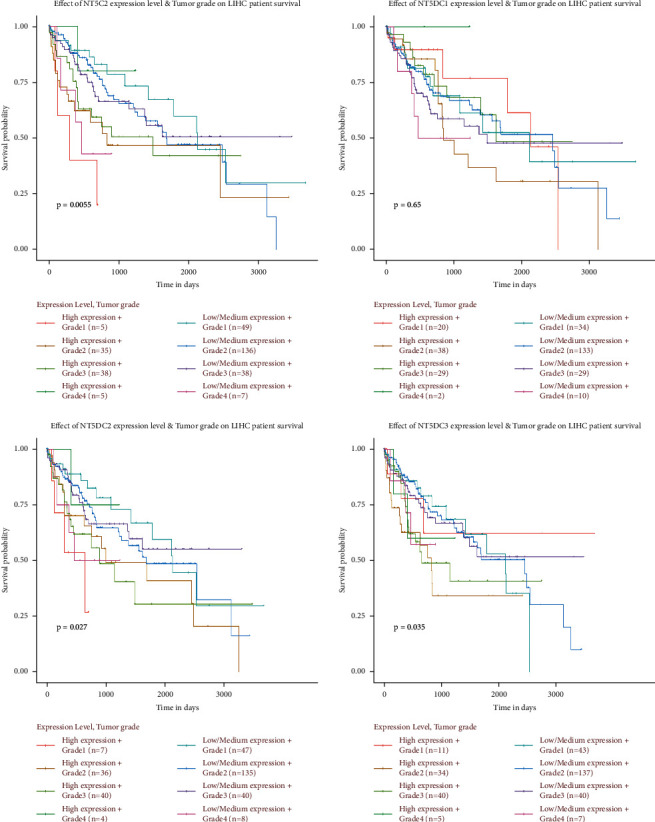
The effect of NT5DC family members' expression for the tumor grade on patient survival outcome with HCC.

**Figure 5:**
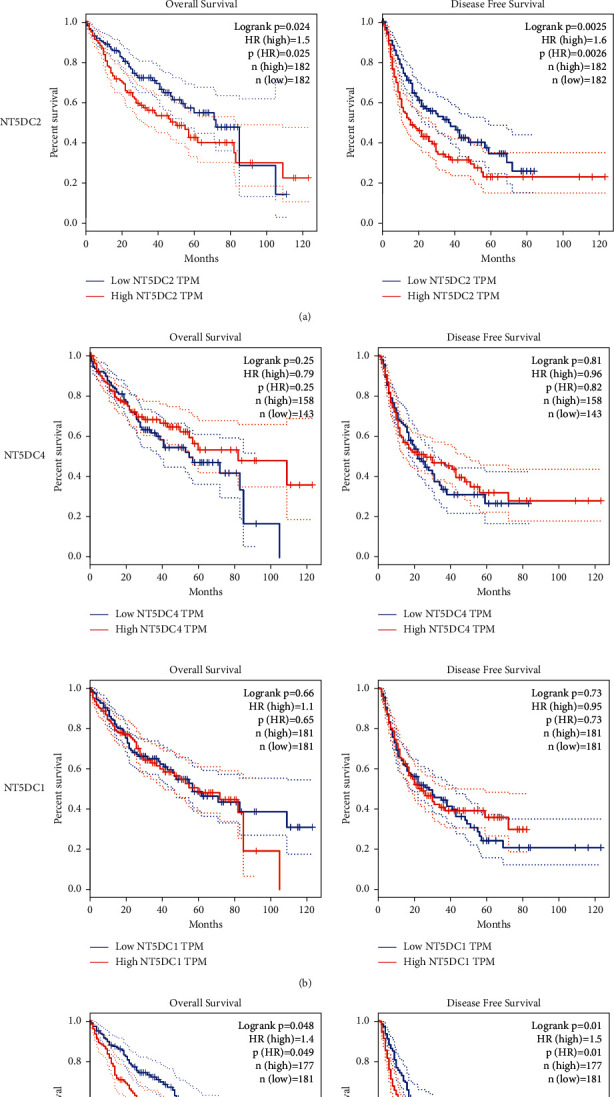
The prognostic significance of NT5DC family members in HCC (GEPIA). OS, overall survival. DFS, disease-free survival.

We used ULCAN to determine the correlation between NT5DC family members' expression and liver tumor grade stage (Figure 4). We observed that the expression of NT5C2 (*P*=0.0055), NT5DC2 (*P*=0.027), and NT5DC3 (*P*=0.035) was closely correlated with tumor stage, suggesting that the expression of NT5C2, NT5DC2, and NT5DC3 increased significantly with the progression of HCC. Furthermore, we used GEPIA to analyze the prognostic value of NT5DC family members in HCC. The survival curve was presented in Figure 5. High expression of NT5C2 (*P*=0.028), NT5DC2 (*P*=0.024), and NT5DC3 (*P*=0.048) predicted poor OS. In addition, high expression of NT5DC2 (*P*=0.0025) and NT5DC3 (*P*=0.01) was associated with poor disease-free survival (DFS).

**Figure 6:**
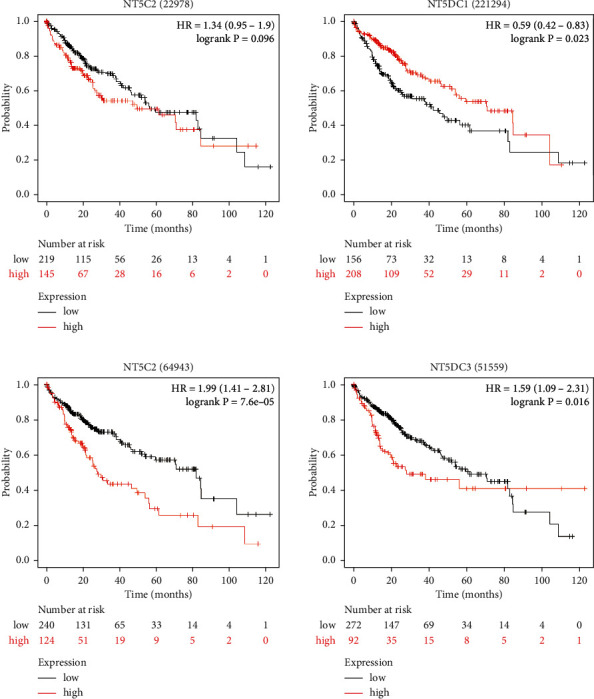
The prognostic value of NT5DC family members in HCC (Kaplan–Meier plotter).

Kaplan–Meier plotter was used to further analyze the relationship between the NT5DC family and HCC patient prognosis (Figure 6). High NT5DC1 (HR: 0.59, 95 CI%: 0.42–0.83, *P*=0.0023) represented good OS. However, high NT5DC2 (HR: 1.99, 95 CI%:1.41–2.81, *P*=7.6*E* − 5) and NT5DC3 (HR: 1.59, 95 CI%: 1.09–2.31, *P*=0.016) were significantly associated with poor OS.

### 3.3. Genetic Alteration and Coexpression of the NT5DC Family in HCC

**Figure 7:**
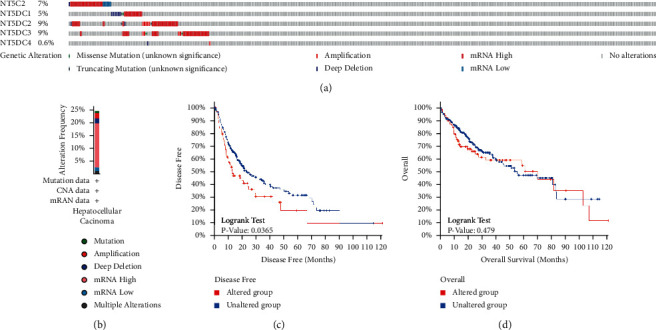
The mutation of NT5DC family members and survival analysis in HCC (cBioPortal). (a, b) Summary of alterations in NT5DC family members in HCC. (c, d) Analysis of OS and DFS in HCC patients with/without genetic changes in NT5DC family members.

The mutation of the NT5DC family in HCC was analyzed by cBioPortal. As shown in Figure 7(a), gene mutation rates of NT5C2, NT5DC1, NT5DC2, NT5DC3, and NT5DC4 in HCC were 7%, 5%, 9%, 9%, and 0.6%, respectively. Most patients had one or more alterations (Figure 7(b)). Furthermore, we evaluated the association between NT5DC family gene mutation and HCC patient prognosis. The results displayed that NT5DC family gene mutation was associated with adverse DFS (*P*=0.0365) (Figure 7(c)). However, there was no significant correlation between NT5DC family gene mutation and OS (*P*=0.479) (Figure 7(d)).

**Figure 8:**
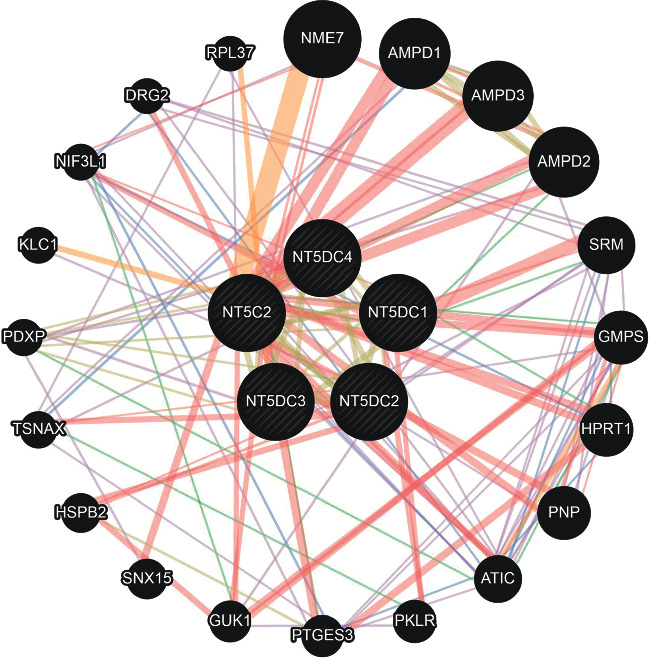
Gene-gene network of NT5DC family members in HCC (GeneMANIA).

We used GeneMANIA to search for genes related to the function of the NT5DC family to explore the regulatory mechanism of the NT5DC family in HCC (Figure 8). We found that the main 20 genes, such as NME7 AMPD1, AMPD2, AMPD3, SRM, GMPS, HPRT1, PNP, ATIC, PKLR, PTGES3, GUK1, SNX15, HSPB2, TSNAX, PDXP, KLC1, NIF3L1, DRG2, and RPL37, were associated with differential expression of the NT5DC family.

### 3.4. Functional Enrichment Analysis of the NT5DC Family in HCC

**Figure 9:**
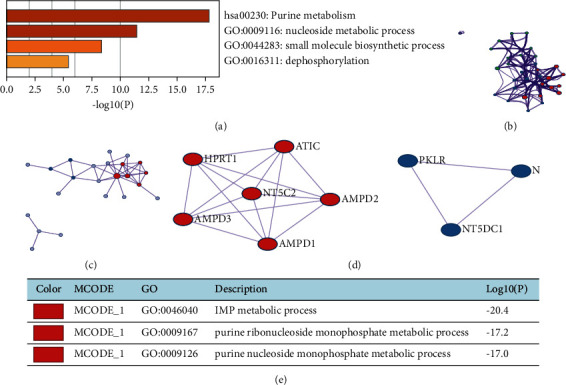
The enrichment analysis of NT5DC family members and the 20 related genes in HCC (Metascape). (a) Four enriched terms for NT5DC family members and the 20 most coexpressed genes. (b) Network of GO enriched terms colored by cluster ID. (c–e) PPI network and MCODE components' analysis.

The Metascape database was used to analyze the biological functions of the NT5DC family and top 20 related genes. The results revealed that the four most related enriched terms included the purine metabolism, nucleoside metabolic process, small molecule biosynthetic process, and dephosphorylation (Figure 9(a)). We also carried out the network of enriched terms colored by ID (Figure 9(b)). In addition, PPI Network and mCODE analysis were implemented. The results showed that the main biological processes involved the IMP metabolic process, purine ribonucleoside monophosphate metabolic process, and purine nucleoside monophosphate metabolic process (Figures 9(c)‐9(e).

### 3.5. Immune Infiltration of the NT5DC Family in HCC

**Figure 10:**
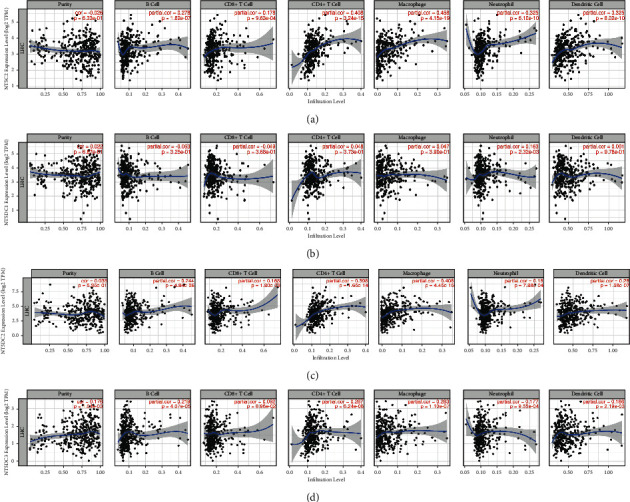
Association between NT5DC family members and immune infiltration in HCC (TIMER). (a) The correlation between NT5C2 and immune infiltration. (b) The correlation between NT5DC1 and immune infiltration. (c) The correlation between NT5DC2 and immune infiltration. (d) The correlation between NT5DC3 and immune infiltration.

We observed that NT5C2 expression was correlated with infiltration of B cells (cor = 0.278, *P*=1.62*e* − 07), CD8+ T cells (cor = 0.178, *P*=9.63*e* − 04), CD4+ T cells (cor = 0.408, *P*=3.24*e* − 15), macrophages (cor = 0.458, *P*=4.14*e* − 19), neutrophils (cor = 0.325, *P*=6.10*e* − 10), and dendritic cells (cor = 0.325, *P*=8.02*e* − 10). NT5DC1 expression was related to neutrophil infiltration (cor = 0.163, *P*=2.32*e* − 3). NT5DC2 expression and infiltration of B cells (cor = 0.244, *P*=4.84*e* − 06), CD8+ T (cor = 0.168, *P*=1.80*e* − 03), CD4+ T cells (cor = 0.408, *P*=4.45*e* − 15), neutrophils (cor = 0.18, *P*=7.88*e* − 04), and dendritic cells (cor = 0.28, *P*=1.58*e* − 07) was positively correlated. NT5DC3 expression and infiltration of B cells (cor = 0.219, *P*=4.07*e* − 05), CD4+ T cells (cor = 0.287, *P*=6.24*e* − 08), macrophages (cor = 0.283, *P*=1.10*e* − 07), neutrophils (cor = 0.177, *P*=9.95*e* − 04) and dendritic cells (cor = 0.166, *P*=2.19*e* − 03) were closely related. The results are presented in Figure 10.

## 4. Discussion

Our study was the first research to analyze the expression, prognosis, mutation, and immune infiltration of the NT5DC family members in HCC based on the online databases. Our findings showed that NT5C2, NT5DC2, and NT5DC3 expression was associated with higher tumor pathologic stage and poor OS. In addition, NT5DC2 and NT5DC3 expression also represented poor DFS. Many studies showed that NT5C2, NT5DC2, and NT5DC3 played an important role in tumor development. It was reported that AML patients with high NT5C2 expression had shorter OS and DFS [20]. It was also found that lung cancer patients with high NT5C2 expression had poor prognosis, and the reduction of NT5C2 could increase the sensitivity to gemcitabine [21]. Another study displayed that silencing NT5C2 increased oxidative metabolism and reduced lung cancer cell proliferation by activating p53 and AMPK [22]. In glioblastoma, NT5C2 could regulate tumor cell proliferation and drug sensitivity [23]. In addition, the activity of NT5C2 was essential for survival in astrocytoma cells [24]. A study found that NT5DC2 was highly expressed in lung cancer, and overexpression of NT5DC2 promoted the proliferation, migration, and invasion of lung cancer cells [25]. On the contrary, NT5DC2 knockdown inhibited cell proliferation and induced cell apoptosis [25]. Zhu et al. showed that NT5DC2 participated in colorectal cancer progression through the VEGF/CCL2 axis, and NT5DC2 was identified as a prognostic marker of colorectal cancer [26]. Guo et al. revealed that NT5DC2 can upregulate Fyn expression and promote glioblastoma progression [27]. They suggested that NT5DC2 may be a promising therapeutic target for glioblastoma. Hu et al. revealed that deletion of NT5DC2 significantly reduced the expression of cyclin B1, cyclin A2, cyclin E1, and CDK1 and suppressed leiomyosarcoma tumour cell proliferation [9]. There was almost no research about the correction between NT5DC 3 or NT5DC4 and tumors. Therefore, the specific mechanism of NT5DC family members in HCC was not well studied.

We further analyzed genes related to the NT5DC family. We found that the top 20 genes were closely related to the NT5DC family, such as AMPD1, AMPD2, and AMPD3. AMPD is involved in nucleotide metabolism [28]. Studies showed that the expression level of AMPD in HCC patients was significantly higher [29]. In addition, we further carried out enrichment analysis. The results displayed that the NT5DC family and its related genes were mainly involved in the IMP metabolic process, purine ribonucleoside monophosphate metabolic process, and purine nucleoside monophosphate metabolic process. In the process of cell canceration, nucleotide metabolism also changes significantly [30]. The NT5DC family was considered to be closely related to nucleotide metabolism [31]. These results indicated that the NT5DC family was potentially carcinogenic.

As an important component of the tumor microenvironment, immune cells play an important role in shaping and forming the tumor microenvironment. Studies have shown that immune cells in the tumor microenvironment act as tumor suppressors or tumor promoters [32]. B cells are an important component of humoral immunity. Studies have found that B cells can not only exert antitumor immunity but also lead to immune tolerance [33]. CD8+ T cells play an important role in antitumor immunity, and their high expression should be beneficial to the prognosis of tumor patients [34]. Neutrophils and dendritic cells are the important participants in tumor immunity and have two sides in tumor progression [35, 36]. CD4+ T cells mainly play a supporting role in the immune system. CD4+ CD25+ regulatory T cells are a subgroup of CD4+ T cells, which play a special role in tumor immune escape [37]. Depletion of CD4+ CD25+ regulatory T cells may enhance the antitumor immunity [38]. There were different opinions on the effect of CD4+ CD25+ regulatory T cells on the prognosis of cancer patients. Curiel et al. believed that tumor-infiltrating regulatory T cells were associated with a high death hazard and reduced survival [39]. A meta-analysis showed that tumor-infiltrating Foxp3+ regulatory T cells in patients with breast cancer predicted poor recurrence-free survival [40]. In hepatocellular carcinoma, many scholars also pointed out that the increase of regulatory T cells indicated a poor prognosis [41, 42]. However, Luo et al. found that, in patients with advanced malignant tumors treated with apatinib, the increase of CD4+ CD25+ regulatory T cells in the blood predicted a longer progression-free survival [43]. Seminerio et al. suggested that a high infiltration of regulatory T cells was associated with longer recurrence-free and overall survivals [44]. Yamamoto et al. proved that pancreatic cancer patients with a higher percentage of CD4+ CD25+ regulatory T cells had survived longer [45]. Interestingly, a study revealed that regulatory T cells' presence in the peripheral blood had no impact on survival for colorectal cancer patients [46]. A previous study showed that NT5DC2 deletion obviously reduced the tumor-associated macrophage (TAM) recruitments through suppressing CCL2/CCR2 and AKT/NF-*κ*B signaling pathways in colorectal cancer [26]. At present, no study has analyzed the relationship between the NT5DC family and tumor immunity in HCC. Our results suggested that NT5DC family expression may be related to B cells, CD8+ T cells and CD4+ T cells, macrophages, neutrophils, and dendritic cell infiltration. These results showed that the expression of the NT5DC family also affected the immune status of HCC patients. These findings provided us with a better understanding of the immune microenvironment of HCC.

The study had some obvious flaws. We relied on online databases for all our analysis. In the future, we would use basic research to further validate these results.

## 5. Conclusions

We demonstrated that NT5DC family expression was closely related to HCC prognosis and immune infiltration. It was revealed that NT5DC family members not only affected the HCC patients prognosis but also changed the immune status of HCC patients. Our findings provided a basis and new insights for further research on the biological function of the NT5DC family in HCC.

## Data Availability

The datasets presented in this study can be found in online repositories. The names of the repository/repositories and accession number(s) can be found in the article.
